# The association between oral health and mild cognitive impairment in community-dwelling older adults

**DOI:** 10.3389/fpubh.2024.1464439

**Published:** 2024-10-17

**Authors:** Niansi Ye, Bei Deng, Hui Hu, Yating Ai, Xueting Liu, Shi Zhou, Yucan Li

**Affiliations:** ^1^School of Nursing, Hubei University of Chinese Medicine, Wuhan, China; ^2^Nursing Department, Wuhan No.1 Hospital, Wuhan, China; ^3^Engineering Research Center of TCM Protection Technology and New Product Development for the Elderly Brain Health, Ministry of Education, Wuhan, China; ^4^Hubei Shizhen Laboratory, Wuhan, China

**Keywords:** mild cognitive impairment, oral health, oral health-related quality of life, older adults, association

## Abstract

**Background:**

Older adults with cognitive impairment can experience poor oral health due to reduced self-care ability, yet the impact of various oral health indicators on the cognitive ability remains unclear. We investigated the relationship between oral health indicators and mild cognitive impairment (MCI) in older adults.

**Methods:**

A cross-sectional study of 234 older adults aged 65 years or over was performed form January to March 2023 at health screening departments of hospitals. This study used the Mini-mental State Examination (MMSE), Montreal Cognitive Assessment (MoCA), Activities of Daily Living (ADL), Clinical Dementia Rating (CDR), and Hachinski Ischemic Score (HIS) to measure MCI. Two qualified dentists performed clinical oral examinations (number of teeth lost, dental caries, removable dentures, periodontitis). The other oral health status was measured by subjective assessment questionnaires, and the oral health-related quality of life (OHRQoL) was assessed by Geriatric Oral Health Assessment Index (GOHAI).

**Results:**

Of the 234 older adults, 166 had MCI and 68 had normal cognitive ability. The univariate analyses revealed that older adults with poor oral health indicators of dental caries, mastication ability, oral and maxillofacial pain, self-perceived oral health status and OHRQoL had lower cognitive levels. The stepwise logistic regression analysis observed that higher education level (OR =  0.06, 95%CI = 0.007, 0.567) and OHRQoL score (OR = 0.92, 95%CI = 0.878, 0.963) were negatively associated with the presence of MCI. The area under the ROC curve (AUC) of MCI was 0.675 (95% CI: 0.600, 0.749) with a low sensitivity of 41.6% and a moderate specificity of 86.8%.

**Conclusion:**

OHRQoL was found to be associated with MCI, implying that OHRQoL may be important in cognitive decline. The GOHAI scale can be used to more easily assess the oral health of older adults, which is important for the timely detection of poor oral status to delay cognitive decline.

## Introduction

Population aging is currently a significant concern facing the world, and with increasing aging, the health problems of older adults constantly highlighted (1). Among the diseases that endanger the health of older adults, dementia has become the fourth leading cause of death after cardiovascular disease, cancer, and stroke (2). China is one of the fastest-aging countries (3), with a prevalence of 5.56% for dementia and 10–20% for mild cognitive impairment (MCI) in adults over 65 years of age (4, 5), which will bring a serious burden to families and society.

MCI is an intermediate state between normal cognition and dementia in which one or more cognitive domains are impaired and do not yet meet the diagnostic criteria for dementia and do not interfere with the ability to perform daily activities (6). The risk of MCI turning into dementia is much higher than normal older adults, with 10–15% of MCI patients developing dementia every year (7). However, to date there is no cure for dementia (8), the stage of MCI provides a critical “window of opportunity” for the prevention and treatment of dementia (9). Early intervention at the MCI stage can effectively delay the progress of dementia, and even restore the older adults to normal cognitive state (10). Therefore, early intervention and risk management for MCI is greatly important and has become a research hot topic at home and abroad, with important academic significance and lucrative socio-economic benefits.

Oral health is a key indicator of overall health, well-being and quality of life, and poor oral health causes millions of people to suffer from devastating pain and increases the financial burden for society (11). The risk of oral diseases increases with age and can affect the cognitive function of older adults (12, 13). Tooth loss, dental caries, mastication ability, and periodontitis are common oral problems. One of the most common oral problems associated with cognitive impairment is tooth loss (14–16). The older adults with tooth loss can lead to nutritional problems such as impaired intake of micronutrients and vitamins, which can lead to affect cognitive function (17). Mastication is a predictor of cognitive impairment, with the ability to masticate decreasing with age and increasing the risk of cognitive impairment (18). The reason is that normal mastication maintains peripheral sensory input, increases blood supply to different areas of the brain, effectively transmits a large amount of sensory information to the brain, and maintains normal learning and memory functions (19). Periodontitis is also associated with cognitive decline, and the inflammatory response it induces can negatively affect the brain (20).

Although the relationships between tooth loss, mastication ability, and cognitive function have been studied, the measurements and results have been heterogeneous. The relationship between other oral health assessment indicators such as dental caries, lateral mastication, and cognitive function are poorly reported. The relationship between OHRQoL and cognitive function is unclear. Moreover, no study has included comprehensive oral health indicators in the analysis of association between the oral health and cognitive function. Information on the association between various aspects of oral health and cognitive function remains insufficient. Accordingly, this study aimed to determine whether there are associations between various aspects of oral health and cognitive function through oral examinations and electronic questionnaire assessments. The innovation of this study lies in the inclusion of comprehensive indicators and the innovative exploration of the subject by using two methods of oral examinations and questionnaire survey.

## Methods

### Design and participants

The sample size was calculated with reference to the preliminary study and based on the sample size calculation formula (21). The sample size was estimated using the following formula. *n* = *Z*^2^ * *p* (1 − *p*)/*e*^2^, *Z* = 1.96, p = prevalence of MCI in Wuhan (87.8%), e = error rate = 0.05; n = 165 patients, with 15% attrition rate, making a total of 190 participants (22). Eligible participants were recruited from health screening departments at two hospitals, part of the Wuhan in China from January to March 2023. The inclusion criteria were age ≥ 65 years, having sufficient visual and auditory discrimination to undergo neuropsychological tests, willingness to participate in the study, and signing the informed consent form. Exclusion criteria were participants with mental disorders and other serious physical illnesses. The study protocol was reviewed and approved by the Medical Ethics Committee of Hubei University of Chinese Medicine (Approved No. of ethic committee: 2019-IEC-003). Participants signed the informed consent form, after due clarification concerning the study and before data collection. This study developed a data web platform specifically to screen and intervene with MCI, including detailed demographic information, neuropsychological tests, and Traditional Chinese Medicine (TCM) technical interventions, enabling direct target population identification while ensuring correct data entry and export.

### Assessments of cognitive function

The cognitive function tests were developed according to the MCI diagnostic criteria of the 2018 China Dementia and Cognitive Disorders Diagnostic and Treatment Guidelines (23). To maximize measurement precision, the cognitive function tests comprised the following tests. The participant was diagnosed with “mild cognitive impairment” when all tests were met.

#### The mini-mental status examination

The MMSE is one of the most widely used cognitive screening tool worldwide and is used to assess the general cognitive function of participants (24). The cut-off values for this tool are related to education level and this study used education-based cut-offs for MMSE in Chinese to exclude definite dementia: illiterate, ≥17 points; primary school, ≥20 points; and junior high school and above, ≥24 points (25). The Cronbach’s *α* of MMSE is 0.825, which has good reliability.

#### The Montreal cognitive assessment

The MoCA is a 30-point test given in 10 min to assess various cognitive tasks including: attention and concentration, executive functions, memory, language, visuoconstructional skills, conceptual thinking, calculations, and orientation. A total MoCA score of 26 is the cut-off value for MCI and older adults with a score of <26 were included in the study (26). The Cronbach’s *α* of MoCA is 0.818, which has a good measurement characteristic.

#### Activities of daily living

This scale is commonly used to assess physical function and is a common indicator of the lives of people with dementia. In this study, the scale was used to exclude people with impairments in daily living skills and participants with scores <16 were included in the study (27). The scale has good reliability and validity, and the Cronbach’s *α* of ADL is 0.966.

#### The clinical dementia rating

The CDR scale comprises six domains: memory, orientation, judgment and problem solving, community affairs, home and hobbies, and personal care, which is graded on a 5-point scale. MCI is defined as CDR = 0.5 (28). The reliability of the scale is 0.84.

#### Hachinski ischemic score

To exclude cognitive decline due to vascular dementia, participants were asked to have a HIS score of ≤4 in the study (29).

### Assessments of oral health status

#### Oral examinations

Two well-trained dentists used probes and mouth mirrors under artificial lights to examine the number of teeth lost, the presence of dental caries, removable dentures, and periodontitis in older adults.

#### Oral health surveys

Geriatric Oral Health Assessment Index (GOHAI), the GOHAI is a scale developed specifically to assess oral health-related quality of life (OHRQoL), which is a 12-item questionnaire with the following response options: 1 = very often; 2 = fairly often; 3 = occasionally; 4 = hardly ever; and 5 = never, including 3 subscales of physical function, pain or discomfort, and psychosocial function. A higher total GOHAI score indicates a better OHRQoL, with a range of 12 to 60. The total score was divided into 3 levels: high (57–60 points), medium (51–56 points), and low (≤50 points) (30, 31). For this study, instead of other measurements, GOAHI was chosen, as it has been the most commonly used measurement tool for evaluating the OHRQoL of the older adults in the world. The Cronbach’s *α* of GOHAI is 0.81, which has a good measurement characteristic. In addition, the caregivers obtained oral health conditions such as mastication ability, unilateral mastication, and oral and maxillofacial pain through interviews.

### Potential covariate assessment

The following potential confounders were examined. As possible parameters associated with MCI, demographic confounders included age, gender, monthly income, and educational level.

### Data analysis

In the descriptive analysis, categorical and continuous variables were shown by frequency, percentage, mean, and deviation. The comparison analysis between the MCI and normal groups was explored using the chi-squared test and fisher’s exact test. If a *p* value was less than 0.05, it was considered statistical significance. Logistic regression was set up using those variables that were found to have a significant difference between cognitive function groups in the univariate analysis as independent variables and MCI (Yes = 1, No = 0) as dependent variables to analyze the relationship between OHRQoL and MCI. The binary logistic regression analysis was performed using the stepwise forward method to control for potential confounders. The odds ratio (OR) and 95% confidence interval (CI) were calculated. Receiver operating characteristic (ROC) curves were used to determine the ability of GOHAI score in identifying MCI. All analyses were conducted using SPSS version 25.0 (IBM Corp., Armonk, NY, United States).

## Results

### Comparison of participant characteristics

This research included 234 older adults, 80 male and 154 female with a mean age of 71.18 ± 4.97 years. The result showed that 65.8% of older adults had a monthly income of ≤3,999, and the education level was concentrated on junior and high school. Based on the assessment criteria for MCI, more than 70% of people had MCI. Of these, 154 were female and 80 were male, women being more likely to have the condition than men (*p* = 0.019). In addition, the data showed that there was an association between MCI and different economic levels (*p* = 0.048) and education levels (*p* < 0.001). MCI is more common in older adults with low-income (73.4%), and low education level (90.0%), but less common in older adults with high-income levels and high education level. In terms of oral health, dental caries (*p* = 0.044), mastication ability (*p* = 0.022), oral and maxillofacial pain (*p* = 0.042), and self-perceived oral health status (*p* = 0.002) all differed across cognitive status. MCI is more common in older adults with dental caries (76.9%), masticatory disorders (>70.0%), oral and maxillofacial pain (>60.0%), and bad self-perceived oral health status (>70.0%) (Table 1).

**Table 1:** Comparison of participants’ characteristics in MCI vs. normal group.

Variable	Normal (*n* = 68)	Mild cognitive impairment (*n* = 166)	Total	*p*-value
Age (years), mean ± SD	71.06 ± 9.53	71.23 ± 7.61	71.18 ± 4.97	0.612
65–69	27 (26.7%)	74 (73.3%)	10 (43.2%)	
70–74	29 (33.3%)	58 (66.7%)	87 (37.2%)	
75–79	8 (30.8%)	18 (69.2%)	26 (11.1%)	
80–84	4 (26.7%)	11 (73.3%)	15 (6.4%)	
≥85	0 (0.0%)	5 (100.0%)	5 (2.1%)	
Gender				0.019*
Male	31 (38.8%)	49 (61.3%)	80 (34.2%)	
Female	37 (24.0%)	117 (76.0%)	154 (65.8%)	
Monthly income (RMB)				0.048*
≤3,999	41 (26.6%)	113 (73.4%)	154 (65.8%)	
4,000–5,999	18 (28.1%)	46 (71.9%)	64 (27.4%)	
6,000–7,999	7 (50.0%)	7 (50.0%)	14 (6%)	
≥8,000	2 (100.0%)	0 (0.0%)	2 (0.9%)	
Education level				<0.001**
Illiteracy	1 (10%)	9 (90%)	10 (4.3%)	
Primary school	1 (7.7%)	12 (92.3%)	13 (5.6%)	
Junior high school	16 (18.4%)	71 (81.6%)	87 (37.2%)	
High school	22 (27.8%)	57 (72.2%)	79 (33.8%)	
Undergraduate and above	28 (62.2%)	17 (37.8%)	45 (19.2%)	
Number of teeth lost				0.921
0	9 (30.0%)	21 (70.0%)	30 (12.8%)	
1–5	35 (29.7%)	83 (70.3%)	118 (50.4%)	
6–10	10 (24.4%)	31 (75.6%)	41 (17.5%)	
>10	14 (31.1%)	31 (68.9%)	45 (19.2%)	
Dental Caries				0.044*
Yes	28 (21.3%)	93 (76.9%)	166 (51.7%)	
No	40 (35.4%)	73 (64.6%)	113 (48.3%)	
Removable dentures				0.771
Yes	41 (30.1%)	95 (69.9%)	136 (58.1%)	
No	27 (27.6%)	71 (72.4%)	98 (41.9%)	
Periodontitis				0.296
Yes	21 (24.4%)	65 (75.6%)	86 (36.8%)	
No	47 (31.8%)	101 (68.2%)	148 (63.2%)	
Mastication ability				0.022*
Very good	6 (75.0%)	2 (25.0%)	8 (3.4%)	
Good	22 (32.4%)	46 (67.6%)	68 (29.1%)	
Moderate	24 (30.8%)	54 (69.2%)	78 (33.3%)	
Bad	15 (20.3%)	59 (79.7%)	74 (31.6%)	
Very bad	1 (16.7%)	5 (83.3%)	6 (2.6%)	
Lateral mastication				0.648
Never	14 (35.0%)	26 (65.0%)	40 (17.1%)	
Rarely	18 (30.0%)	42 (70.0%)	60 (25.6%)	
Occasionally	11 (35.5%)	20 (64.5%)	31 (13.2%)	
More often	17 (23.9%)	54 (76.1%)	71 (30.3%)	
Frequently	8 (25.0%)	24 (75.0%)	32 (13.7%)	
Oral and maxillofacial pain				0.042*
Never	26 (37.1%)	44 (62.9%)	70 (29.9%)	
Rarely	24 (27.6%)	63 (72.4%)	87 (37.2%)	
Occasionally	16 (31.4%)	35 (68.6%)	51 (21.8%)	
More often	2 (7.7%)	24 (92.3%)	26 (11.1%)	
Frequently	0 (0.0%)	0 (0.0%)	0 (0.0%)	
Self-perceived oral health status				0.002*
Very good	5 (71.4%)	2 (28.6%)	7 (3.0%)	
Good	27 (41.5%)	38 (58.5%)	65 (27.8%)	
Moderate	20 (26.3%)	56 (73.7%)	76 (32.5%)	
Bad	16 (20.5%)	62 (79.5%)	78 (33.3%)	
Very bad	0 (0.0%)	8 (100.0%)	8 (3.4%)	

n, number; SD, standard deviation; RMB, Chinese yuan; * < 0.05.; ** < 0.01.

### The correlation between GOHAI scale scores and MCI

The mean GOHAI assessment index score in this study was 47.00 ± 7.958, which was at a low level of oral health status. The mean score of 14.46 ± 3.501 for the physical function dimension, 11.12 ± 2.336 for the pain and discomfort dimension and 21.35 ± 3.138 for the psychosocial dimension, indicating that OHRQoL of older adults in the region is poor and needs to be improved. We also found a statistically significant weak negative correlation between GOHAI test scores and MCI (*p* < 0.001). The GOHAI scores were higher in older adults with normal cognition than in those with MCI, suggesting that MCI in older adults is associated with poorer OHRQoL (Table 2).

**Table 2:** The correlation between GOHAI scale scores and MCI.

Variable	Normal (*n* = 68) Mean ± SD	Mild cognitive impairment (*n* = 166) Mean ± SD	Total (*n* = 234) Mean ± SD	*r*	*p*-value
Physiological functions	15.72 ± 3.407	13.98 ± 3.395	14.46 ± 3.501	−0.228	<0.001
Pain and discomfort	11.88 ± 2.263	10.81 ± 2.308	11.12 ± 2.336	−0.208	0.001
Psychosocial functions	22.65 ± 2.520	20.89 ± 3.134	21.35 ± 3.138	−0.261	<0.001
Total score	50.25 ± 6.933	45.67 ± 7.987	47.00 ± 7.958	−0.262	<0.001

r, correlation coefficient; n, number; SD, standard deviation.

### Oral health-related risk factors for MCI

Table 3 describes binary logistic regression analysis for MCI with risk factors. Treating MCI and normal cognition as dependent variables, the variables that were significant for the above analysis (gender, monthly income, education level, dental caries, mastication ability, oral and maxillofacial pain, self-perceived oral health status, and total GOHAI score) were selected as independent variables to enter into a binary logistic regression analysis. Table 3 shows that independent risk factors for MCI were education level (OR = 0.06, 95%CI = 0.007, 0.567) and OHRQoL (OR = 0.92, 95%CI = 0.878, 0.963). The higher education level reduced the risk of MCI, while the lower OHRQoL increased the risk of MCI.

**Table 3:** Binary logistic regression analysis for MCI with risk factors.

Variable	*B*	SE	Odds ratio (95% CI)	*p-*value
**Education level**				
Primary school	0.242	1.498	1.27 (0.068 ~ 23.992)	0.872
Junior high school	−0.840	1.105	0.43 (0.049 ~ 3.764)	0.447
High school	−1.241	1.099	0.29 (0.034 ~ 2.489)	0.259
Undergraduate and above	−2.753	1.115	0.06 (0.007 ~ 0.567)	0.014
GOHAI score	−0.084	0.023	0.92 (0.878 ~ 0.963)	<0.001

B, unstandardized regression coefficient; SE, standard error; CI, confidence interval.

### ROC analyses of GOHAI score in identifying MCI

We also carried out the ROC analyses to verify the accuracy and sensitivity of GOHAI in identifying MCI. The best threshold value of GOHAI score in identifying MCI was 43.5. The area under the ROC curve (AUC) of MCI was 0.675 (95%CI: 0.60, 0.75) with a low sensitivity of 41.6% and a moderate specificity of 86.8%.

## Discussion

The World Health Organization has listed oral health as one of the top 10 criteria for human health. At present, as the transformation of the medical pattern and the concept of Healthy China continues to grow, oral health has become an important part of people’s pursuit of health. The fourth national oral health epidemiological survey in China showed that the current oral health status of older adults in China is not optimistic, with the serious prevalence of dental caries and periodontal disease (32), and these poor oral health status may increase the risk of cognitive impairment in older adults.

However, research on the relationship between oral health status and MCI is currently limited, which is constrained by the conventional views that “dental disease is not a disease” and “losing teeth is normal aging” (33). This study assessed the relationship between oral health indicators and MCI, the results of the subjective and objective examination revealed that gender, education level, economic level, dental caries, mastication ability, oral and maxillofacial pain, self-perceived oral health status, and the OHRQoL were all correlated with cognitive status in older adults. The regression analysis showed that the GOHAI was negatively associated with cognitive status, and poor OHRQoL in older adults was an independent risk factor for MCI. ROC curves revealed the GOHAI score was determined with 41.6% sensitivity and 86.8% specificity in the prediction of MCI at a cut-off value of 43.5. This is the first study to find that poorer OHRQoL is an independent risk factor for MCI. OHRQoL is a reflection of oral health physiological function, pain and discomfort, and psychosocial function and is closely related to oral physiological dysfunction such as tooth loss, dental caries, and decreased mastication ability, which may be an indicator of true oral health problems. Despite the low sensitivity, the study results provide a certain reference to for clinical study.

Oral diseases are the main influencing factors of OHRQoL, and older adults with poor oral examination results have low OHRQoL (34). Oral diseases, including functional tooth loss, dental caries, and poor mastication function can negatively affect OHRQoL. The low OHRQoL predicts oral problems in older adults and may influence cognitive function through physiological effects (35, 36). Moreover, oral diseases damage the harmony and esthetics of the face, which restricts the social activities of the older adults and can have a detrimental effect on their mental health level. Previous studies have shown that poor OHRQoL can lead to loneliness (37), depression (38), and restriction of mobility and social participation (39). Loneliness (40), depression (41), and reduced social activities (42) have been associated with cognitive decline in older adults. The psychological influence of oral health or the negative psychological impact of OHRQoL on older adults can also affect cognitive function. Therefore, identifying and managing OHRQoL in older adults can help to relieve discomfort, improve oral problems, reduce psychological stress, and also improve cognitive status in older adults with MCI.

In the regression analysis, oral problems such as tooth loss, dental caries, and mastication impairment have not been found to be associated with MCI in older adults, which may be related to factors such as the sample size and the assessment method. Obviously, the impact of poor oral health on the OHRQoL of older adults has a negative effect on cognitive function. Oral health is an easily identifiable and modifiable risk factor. The MCI stage, where older adults retain good mobility and can take care of their oral health, is the best stage for interventions for poor oral health in older adults with cognitive disorders. The OHRQoL assess enables the detection of oral problems, which will help to slow down the decline in cognitive function. The regular objective examination of oral health often has special requirements, and GOHAI is a more comprehensive and subjective evaluation system that reflects the new medical model and view of health. The GOHAI can be used by a variety of researchers to assess the OHRQoL and to improve poor oral status, healthcare behaviors, and habits promptly, which is important for the overall health and quality of life of older adults. Therefore medical workers can adopt targeted interventions to create proper oral awareness and establish proper oral habits in older adults, which may improve their OHRQoL and improve cognitive performance.

There are some limitations in our study. First, participants were recruited through hospitals, which may have selection bias. Second, our study was designed as a cross-sectional study, it was difficult to draw conclusions about the causal relationship between oral health status and MCI. Additionally, Some oral health indicators were assessed by self-report, which may be subject to recall bias. In subsequent studies, there is a need to develop strict inspection measures, refine the classification indicators, expand the sample size and reduce confounding factors to further improve the study design, preferably through longitudinal studies to clarify the causal relationship between various oral health indicators and MCI.

## Conclusion

This study intentionally developed a data web platform to screen and intervene older adults with MCI. To improve measurement accuracy and data collation, older adults with MCI were identified through multiple assessments, and their oral health was assessed through subjective and objective examinations. This is the first study to show that OHRQoL is an independent risk factor for MCI and that poor OHRQoL is associated with poor cognitive function in older adults. Therefore medical workers can focus on increasing oral health awareness, establishing proper oral hygiene habits, improving oral problems, and enhancing education about the causes and symptoms of oral problems in older adults, which may improve their OHRQoL and thus maintain and improve cognitive ability. It is also important to pay attention to the psychological impact of poor OHRQoL on older adults, and maintaining the psychological health of older adults also has a role in improving OHRQoL and cognitive ability. However, longitudinal studies with large samples on various oral health indicators are needed in the future.

## Data Availability

The original contributions presented in the study are included in the article, further inquiries can be directed to the corresponding author/s.
